# Predicting dysthyroid optic neuropathy in moderate-to-severe thyroid eye disease: a clinically applicable nomogram

**DOI:** 10.1530/ETJ-25-0226

**Published:** 2025-12-18

**Authors:** Ruolin Hu, Siqi Tang, Xinyu Liu, Zewei Liu, Zhipeng Cui, Jingyue Chen, Yanan Wang, Feixue Jiang, Jingyi Zhu, Chao Wan, Yizhou Sun, Lei Shi, Zheng Wang, Chenyan Li, Xiaohui Yu, Chuyuan Wang, Weiwei Wang, Yaxin Lai, Yanli Cao, Xiaoli Wang, Yushu Li, Zhongyan Shan, Weiping Teng

**Affiliations:** ^1^ Department of Endocrinology and Metabolism, The Institute of Endocrinology, NHC Key Laboratory of Diagnosis and Treatment of Thyroid Disease, The First Hospital of China Medical University, Shenyang, P.R. China; ^2^ Department of Radiology, The First Hospital of China Medical University, Shenyang, P.R. China; ^3^ Department of Ophthalmology, The First Hospital of China Medical University, Shenyang, P.R. China; ^4^ Department of Otorhinolaryngology, The First Hospital of China Medical University, Shenyang, P.R. China

**Keywords:** thyroid eye disease, dysthyroid optic neuropathy, predictive model

## Abstract

**Objective:**

Dysthyroid optic neuropathy (DON) is a severe complication of thyroid eye disease (TED) with limited early detection methods. This study aimed to investigate the clinical characteristics of patients with TED who developed DON and to establish a predictive model for early identification of high-risk cases.

**Methods:**

Herein, 257 TED patients were prospectively included, of whom 68 (26.5%) developed DON. All patients were divided into derivation and validation cohorts, and Least Absolute Shrinkage and Selection Operator (LASSO) regression and logistic regression analyses were applied to identify clinical factors and construct a prediction model.

**Results:**

In the derivation cohort (185 TED patients), 49 (26.5%) developed DON. DON patients showed significantly higher prevalence of pretibial myxedema (PTM) (22.4 vs 5.9%, *P* = 0.001), diabetes mellitus (18.4 vs 7.4%, *P* = 0.029), older age (58.04 ± 11.30 years vs 47.99 ± 10.65 years, *P* < 0.001), higher CAS (5 vs 4, *P* < 0.001), elevated triglyceride (TG) levels (1.44 mmol/L vs 1.15 mmol/L, *P* = 0.042), and lower visual functioning (VF) (43.75 vs 62.50, *P* < 0.001). LASSO regression analysis identified age, PTM, TG, VF, and CAS as independent predictors of DON. The developed nomogram presented AUCs of 0.853 (95% CI: 0.792–0.914) and 0.856 (95% CI: 0.762–0.950) in the derivation and validation cohorts, respectively.

**Conclusions:**

Altogether, the findings of this study identify advanced age, elevated CAS, increased TG, lower VF, and PTM as significant predictors of DON in patients with TED. The proposed nomogram offers a practical clinical tool for risk stratification, providing clinicians with an approach for individualized risk assessment and timely therapeutic intervention.

## Introduction

Thyroid eye disease (TED), also known as Graves’ orbitopathy or thyroid-associated orbitopathy, represents the primary extrathyroidal manifestation of Graves’ disease (GD) and affects approximately 25–40% of patients with GD (1, 2). Reportedly, the incidence of TED is estimated to be 2.67–3.3 and 0.54–0.9 per 100,000 person-years in women and men, respectively (3, 4). The European Group on Graves’ Orbitopathy (EUGOGO) established a standardized severity classification system, grouping TED into the following three categories: mild, moderate-to-severe, and sight-threatening (5). Dysthyroid optic neuropathy (DON) lies in the sight-threatening category, with a 5–8% incidence in patients with TED. Notably, DON has been associated with reduced visual acuity, impaired color vision, and a marked decline in the quality of life (QoL) for those affected (6).

Diagnosing DON presents substantial clinical challenges, primarily owing to the absence of pathognomonic symptoms and the unpredictable progression of TED (7). Presently, diagnostic protocols require the presence of ≥2 characteristic clinical manifestations, corroborated by ancillary testing, with common indicators including impaired color vision and optic nerve head edema (8). Per the EUGOGO guidelines, high-dose intravenous methylprednisolone (IVMP) (0.5–1.0 g) for either consecutively 3 d or on alternate days is recommended. In the case of no improvement or worsening of visual function (VF) after 1 week, or limited improvement after 2 weeks of treatment, urgent orbital decompression is recommended (5). However, present studies report variable response rates (22–61%) to high-dose IVMP therapy in DON patients (9). This highlights the need for accurate prediction of DON occurrence in patients with TED, along with timely identification and intervention for high-risk individuals, for improving the prognosis.

Many risk factors have been implicated in TED and DON progression, including smoking, thyroid dysfunction, older age at onset, hypertrophy of the extraocular muscles, radioiodine therapy, type 2 diabetes mellitus (DM), and dyslipidemia (DLP) (10, 11, 12, 13). Nonetheless, the clinical relevance of these potential risk factors in Chinese TED populations remains unelucidated. This study aimed to evaluate the clinical features of patients with moderate-to-severe TED and DON to ultimately identify DON-associated potential risk factors in this cohort. In addition, a clinically applicable prediction model was established to facilitate early recognition of high-risk cases and prevent therapeutic delays.

## Methods

### Study design and population

This cross-sectional study was conducted at the First Hospital of China Medical University. The study protocol was approved by the Ethics Committee of the First Hospital of China Medical University (approval number: 2025-558-2). Each participant was informed about the project and willingly completed an informed consent document. This study adheres to the guidelines of the Declaration of Helsinki as well as the Transparent Reporting of a Multivariable Prediction Model for Individual Prognosis or Diagnosis reporting (TRIPOD) guidelines (14) and the Strengthening the Reporting of Observational Studies in Epidemiology Statement (STROBE) (15).

We prospectively included patients diagnosed with TED who were admitted to the Department of Endocrinology between November 2020 and February 2025. For each patient, the clinical data and diagnoses were collected at the time of their initial admission for TED. The patients were included in the study if they had: i) a confirmed diagnosis of active (a clinical activity score (CAS) of ≥3) TED; ii) their severity of TED was moderate-to-severe or sight-threatening (only DON was included); iii) no prior glucocorticoid pulse therapy or immunosuppressive treatment for TED within the preceding 3 months. The exclusion criteria were as follows: i) anyone under the age of 18; ii) incomplete data (*n* = 6); iii) patients with mild TED (*n* = 12); iv) patients suffering from viral hepatitis or chronic kidney disease, along with pregnant or lactating women, and those with corneal damage.

Ultimately, 257 eligible TED patients were enrolled. To facilitate the development and validation of the model, the cohort was divided into two groups using a time-based split: a derivation cohort (*n* = 185), including patients admitted before July 16, 2024, and a validation cohort (*n* = 72), comprising patients admitted on or after this date. The number of DON cases was 49 (26.5%) in the derivation cohort and 19 (26.4%) in the validation cohort. The derivation cohort was used to develop the predictive model, whereas the validation cohort was used for subsequent evaluation.

### Ophthalmic assessment

All participants underwent a standardized evaluation conducted by a multidisciplinary team, which consisted of fellowship-trained ophthalmologists, endocrinologists, and neuroradiologists. The diagnosis of TED was established based on the criteria proposed by Bartley *et al.* (16). Disease severity was stratified in accordance with the EUGOGO guidelines, with CAS assessed using the validated 7-point scale (5, 17). The patients were diagnosed with moderate-to-severe TED if they had two or more of the following: i) eyelid retraction measuring 2 mm or greater; ii) moderate or severe soft tissue involvement; iii) exophthalmos at least 3 mm above the norm for their racial and gender group; iv) inconstant or constant diplopia.

The diagnosis of DON required the fulfillment of the following two criteria: i) radiologically confirmed TED with evidence of orbital apex crowding and/or optic nerve compression on magnetic resonance imaging (MRI) or computed tomography (CT); ii) at least two clinical signs of optic neuropathy supported by ophthalmologic auxiliary examinations, with no other identifiable etiology. These signs included: i) a decline in the best-corrected visual acuity (BCVA) of ≥2 lines; ii) relative afferent pupillary defect (RAPD); iii) abnormalities in color vision as assessed using pseudoisochromatic plates; iv) abnormal visual evoked potentials; v) visual field defects as measured using the *Humphrey field analyzer*; vi) optic disc edema, as evaluated using optical coherence tomography (OCT) for objective assessment.

All TED patients underwent the aforementioned ophthalmologic and imaging examinations. If an MRI was not feasible, a CT was performed for detection. Patients who met the diagnostic criteria were diagnosed with DON and enrolled in the DON group. In cases of bilateral TED, the eye with more severe symptoms, as determined by the CAS, was selected as the study eye. For bilateral DON, the eye with the greater vision loss was selected as the study eye.

### Data collection

Patient characteristics included demographic data (including age, sex, smoking status, and a family history of thyroid diseases), endocrine comorbidities (DM, osteoporosis (OP)), and disease-specific variables (duration of Graves’ disease (GD) and TED, presence of pretibial myxedema (PTM), history of thyroid surgery or radioactive iodine therapy (RAI) before initial admission). Clinical assessments consisted of the CAS, disease severity, degree of exophthalmos, diploma score (Gorman score) (18) and the quality of life evaluated by the EUGOGO disease-specific GO-QoL questionnaire, which includes subjective VF score and GO-QoL appearance score (GO-QoL AP) (19).

We evaluated the included lipid profiles (total cholesterol (TC), triglycerides (TG), low-density lipoprotein cholesterol (LDL-C)), vitamin D3 levels, and thyroid function tests (free thyroxine (FT4), free triiodothyronine (FT3), thyroid-stimulating hormone (TSH), thyroid peroxidase antibody (TPOAb), thyroglobulin antibody (TGAb), and TSH receptor antibody (TRAb)).

All ophthalmologic and laboratory examination data used for analysis were collected at the time of initial admission.

### Laboratory measurements

Thyroid function parameters, including FT4, FT3, TPOAb, TGAb, TSH, and TRAb levels, were determined through electrochemiluminescence immunoassay (ECLIA) on Architect i2000SR analyzers (Abbott Laboratories, USA) in strict adherence to the manufacturer’s protocols.

### Statistical analysis

The Shapiro–Wilk test was employed to assess data normality. Continuous variables that adhered to a normal distribution were expressed as means accompanied by standard deviations (SD), whereas the non-normally distributed variables were reported as median (interquartile range, IQR). Between-group comparisons for parametric and non-parametric continuous variables were conducted using Student’s *t*-test and Mann–Whitney U–test, respectively. Categorical variables were reported as frequencies (percentages), and the between-group differences were evaluated using Pearson’s chi-square test or Fisher’s exact test, contingent upon the expected cell frequencies.

All variables were first subjected to univariable logistic regression analysis, keeping those with a *P*-value under 0.1 in the running to ensure we did not miss any potential predictors that might turn out to be important. We employed the Least Absolute Shrinkage and Selection Operator (LASSO) regression to pinpoint potential predictors while eliminating multicollinearity among variables. Subsequent to this, multivariable binary logistic regression was employed to evaluate the independent risk factors. These predictors were incorporated into a clinically applicable nomogram. All LASSO procedures, nomogram construction, and receiver operating characteristic (ROC) curve analyses were conducted using R statistical software (version 4.4.2; R Foundation for Statistical Computing), whereas the other statistical analyses were performed with IBM SPSS Statistics (version 29.0; IBM Corp). Statistical significance was defined as a two-tailed *P* < 0.05.

## Results

### Clinical characteristics of the two study cohorts

**Figure 1:**
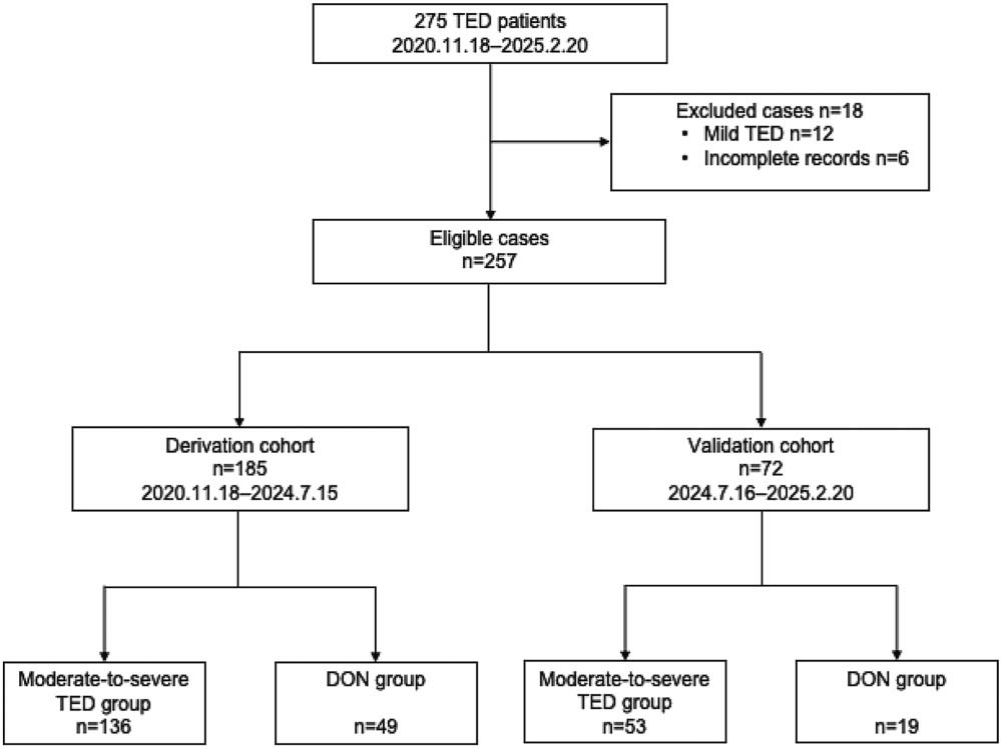
Flow chart for patient selection.

In total, 257 patients with TED were included in this study, with 68 (26.5%) being diagnosed with DON (Fig. 1). The derivation cohort comprised 185 patients (mean age = 50.65 ± 11.67 years). Most participants were female (*n* = 109, 58.9%) and exhibited a high prevalence of GD (*n* = 172, 93.0%). Thyroid disorder management for patients before enrollment was as follows: antithyroid drugs (*n* = 153, 82.7%), levothyroxine (*n* = 15, 8.1%), no medication (*n* = 17, 9.2%), RAI therapy (*n* = 23, 12.4%), and thyroidectomy (*n* = 11, 5.9%). Among them, 19 patients (10.3%) presented with concurrent PTM. Notably, 49 patients developed DON (Table 1, Supplementary Table 1 (see section on Supplementary materials given at the end of the article)), and they were significantly older (58.04 ± 11.30 years vs 47.99 ± 10.65 years, *P* < 0.001), had higher CAS (5 vs 4, *P* < 0.001), and exhibited lower GO-QoL subjective VF score (43.75 vs 62.50, *P* < 0.001) compared with moderate-to-severe TED patients. Comorbidity analysis revealed significantly higher rates of DM (18.4 vs 7.4%, *P* = 0.029), PTM (22.4 versus 5.9%, *P* = 0.001), and OP (18.4 vs8.1%, *P* = 0.047) in the DON group.

**Table 1:** Demographics and clinical characteristics among patients in the derivation cohort. Data are presented as mean ± SD, median (IQR), or as *n* (%). Statistically significant *P*-values are presented in bold.

Characteristics	Total	DON	Moderate to severe TED	*P*-value
*n*	185	49	136	
Age (years)	50.65 ± 11.67	58.04 ± 11.30	47.99 ± 10.65	<0.001*
Sex				0.471^‡^
Female	109 (58.9)	31 (63.3)	78 (57.4)	
Male	76 (41.1)	18 (36.7)	58 (42.6)	
BMI (kg/m^2^)	24.05 (22.05–27.17)	24.98 (22.38–26.30)	24.02 (21.88–27.37)	0.785^†^
Smoking history				0.570^‡^
No	112 (60.5)	28 (57.1)	84 (61.8)	
Yes	73 (39.5)	21 (42.9)	52 (38.2)	
Treatment of thyroid disease				0.803^§^
Antithyroid drugs	153 (82.7)	40 (81.6)	113 (83.1)	
Levothyroxine	15 (8.1)	5 (10.2)	10 (7.4)	
Without medication	17 (9.2)	4 (8.2)	13 (9.6)	
Thyroidectomy	11 (5.9)	2 (4.1)	9 (6.6)	0.771^‡^
RAI	23 (12.4)	6 (12.2)	17 (12.5)	0.963^‡^
Family history of thyroid disease	21 (11.4)	5 (10.2)	16 (11.8)	0.768^‡^
Pretibial myxedema	19 (10.3)	11 (22.4)	8 (5.9)	**0.001^‡^**
Diabetes mellitus	19 (10.3)	9 (18.4)	10 (7.4)	**0.029^‡^**
Osteoporosis	20 (10.8)	9 (18.4)	11 (8.1)	**0.047^‡^**
Dyslipidemia	74 (40)	25 (51.0)	49 (36.0)	0.066^‡^
Duration of TED (month)	7 (4–12)	7 (4–12)	7 (3–12)	0.914^†^
CAS	4 (3–5)	5 (4–5)	4 (3–4)	**<0.001^†^**
Diplopia				0.166^‡^
Yes	121 (65.4)	36 (73.5)	85 (62.5)	
No	64 (34.6)	13 (26.5)	51 (37.5)	
Gorman score				0.051^‡^
No diplopia (0 points)	64 (34.6)	13 (26.5)	51 (37.5)	
Intermittent diplopia (1 point)	45 (24.3)	8 (16.3)	37 (27.2)	
Inconstant diplopia (2 points)	34 (18.4)	14 (28.6)	20 (14.7)	
Constant diplopia (3 points)	42 (22.7)	14 (28.6)	28 (20.2)	
Proptosis (mm)	21 (19–23)	21 (19–24)	21 (19–23)	0.406^†^
GO-QoL				
VF	56.25 (37.50–75.00)	43.75 (28.13–57.74)	62.50 (41.97–85.12)	**<0.001^†^**
AP	56.25 (43.75–81.25)	56.25 (43.75–81.25)	62.50 (43.75–81.25)	0.944^†^
TRAb (IU/L) (RR: 0.00–1.75)	10.65 (4.18–22.52)	12.04 (5.44–31.76)	9.27 (3.86–22.19)	0.151^†^
TG (mmol/L) (RR: 0.00–1.70)	1.20 (0.85–1.83)	1.44 (0.97–2.28)	1.15 (0.83–1.63)	**0.042^†^**
TC (mmol/L) (RR: 0.00–5.72)	4.82 ± 1.06	5.04 ± 1.21	4.74 ± 0.99	0.081*
LDLC (mmol/L) (RR: 0.00–3.64)	2.96 ± 0.89	3.18 ± 1.02	2.89 ± 0.83	0.046*

TED, thyroid eye disease; TRAb, TSH receptor antibody; TC, total cholesterol; TG, triglyceride; LDL-C, low-density lipoprotein cholesterol; RAI, radioactive iodine; RR, reference range; UA, uric acid; GO-QoL, Graves orbitopathy on quality of life; VF, subjective VF score (from GO-QoL); AP, GO-QoL appearance score.

Statistical analysis was performed using:

* Student’s *t*-test.

^†^ Mann–Whitney U test.

^‡^ Chi-square test or corrected Chi-square test.

^§^ Fisher’s exact test.

**Figure 2:**
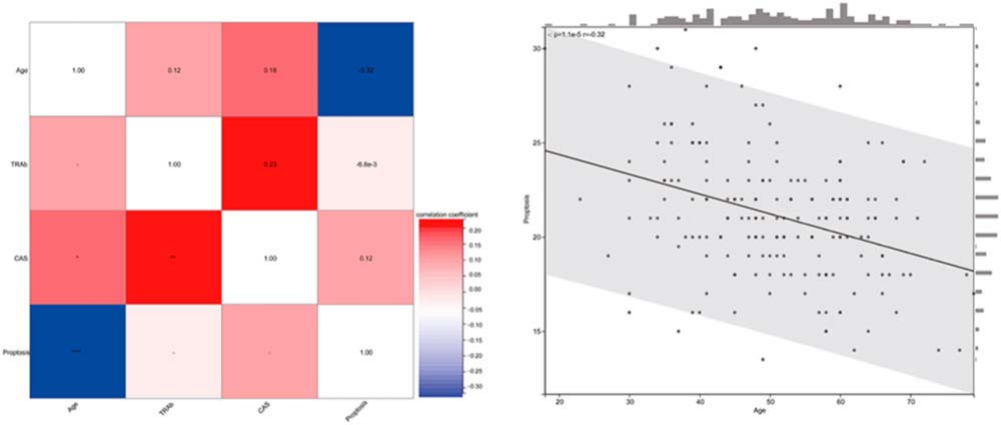
A heatmap was used to perform Spearman correlation analysis between age, proptosis, TRAb, and CAS, and a scatter plot was used to illustrate the linear relationship between age and proptosis.

Notably, patients with DON and those with moderate-to-severe TED presented no significant differences regarding thyroid disease history, antithyroid medication use, RAI treatment history, or thyroidectomy. Similarly, demographic characteristics such as sex distribution, smoking history, and family history of thyroid disease were comparable between the groups. The duration of thyroid disease and TED, the degree of proptosis, diplopia, Gorman score, and GO-QoL AP score showed no significant intergroup differences. However, Spearman correlation analysis revealed that age was significantly negatively correlated with the degree of proptosis (*ρ* = −0.317, *P* < 0.001) in patients with TED. In contrast, TRAb levels were significantly positively correlated with CAS (*ρ* = 0.228, *P* = 0.002) (Fig. 2). Subjective VF score correlated negatively with age (*ρ* = −0.202, *P* = 0.006), CAS (*ρ* = −0.185, *P* = 0.011), and the Gorman score (*ρ* = −0.510, *P* < 0.001).

Moreover, patients with DON exhibited significantly higher levels of TG (1.44 mmol/L vs 1.15 mmol/L, *P* = 0.042) and LDL-C (3.18 ± 1.02 mmol/L vs 2.89 ± 0.83 mmol/L, *P* = 0.046) at initial admission compared with those with moderate-to-severe TED. Notably, no significant intergroup differences were found regarding levels of serum thyroid hormone, TC, or vitamin D3 at the initial visit.

In the validation cohort, among the 72 patients with TED (mean age = 51.22 ± 12.99 years), 19 (26.4%) were diagnosed with DON. Similar to the derivation cohort, most participants were female (*n* = 48, 66.7%) and presented with GD (*n* = 66, 91.7%). Prior thyroid management of patients was as follows: antithyroid drugs (*n* = 64, 88.9%), levothyroxine (*n* = 5, 6.9%), no medication (*n* = 3, 4.2%), RAI therapy (*n* = 4, 5.6%), and thyroidectomy (*n* = 4, 5.6%). Baseline characteristics did not exhibit any significant differences between the derivation and validation cohorts (Table S2). The baseline data of the total population and validation cohort are presented in Supplementary Tables 2 and 3, respectively.

### Factors associated with the presence of DON

**Figure 3:**
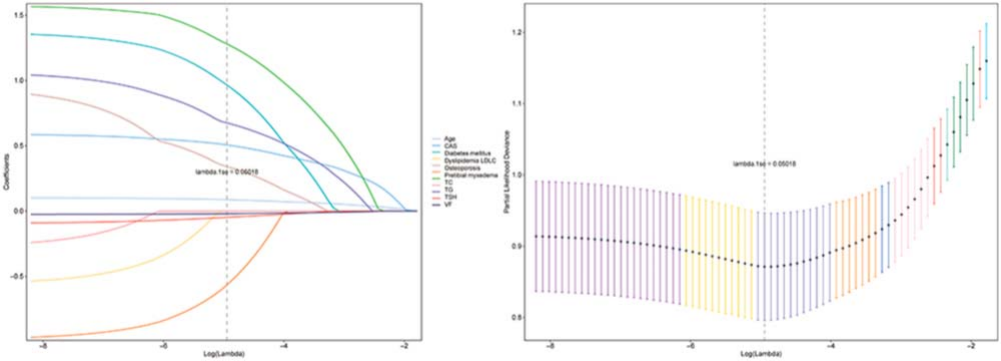
The eleven clinical features were entered into a LASSO regression model, from which five features with non-zero coefficients and a Lambda.1se were selected.

Univariate logistic regression analysis in the derivation cohort identified potential predictors of DON (Table 2). Subsequently, 11 variables (*P* < 0.1) were entered into the LASSO regression analysis. Using ten-fold cross-validation, the optimal penalty parameter (Lambda.1se) was determined, and only variables with non-zero coefficients at this threshold were retained for multivariate analysis (Fig. 3).

**Table 2:** Univariate and multivariate logistic regression analysis of risk factors for DON. Statistically significant *P*-values are presented in bold text.

Factors	Univariable analysis		Multivariable analysis	
	Odds ratio (95% CI)	*P*-value	Odds ratio (95% CI)	*P*-value
Age	1.092 (1.053–1.131)	**<0.001**	1.101 (1.054–1.150)	**<0.001**
Diabetes mellitus	2.835 (1.077–7.465)	**0.035**		
Pretibial myxedema	4.632 (1.738–12.341)	**0.002**	4.085 (1.054–14.342)	**0.028**
Triglycerides	1.552 (1.057–2.279)	**0.025**	2.060 (1.277–3.322)	**0.003**
LDLC	1.452 (1.003–2.101)	**0.048**		
Clinical activity score	1.961 (1.438–2.676)	**<0.001**	1.776 (1.234–2.557)	**0.002**
VF	0.974 (0.959–0.989)	**0.001**	0.980 (0.964–0.996)	**0.014**
Osteoporosis	2.557 (0.989–6.612)	0.053		
TSH	0.896 (0.791–1.016)	0.086		
Total cholesterol	1.318 (0.965–1.800)	0.083		
Dyslipidemia	1.849 (0.995–3.580)	0.068		
TSH receptor antibody	1.007 (0.990–1.025)	0.437		

VF, subjective VF score (from GO-QoL); TSH, thyroid-stimulating hormone; LDLC, low-density lipoprotein cholesterol.

**Figure 4:**
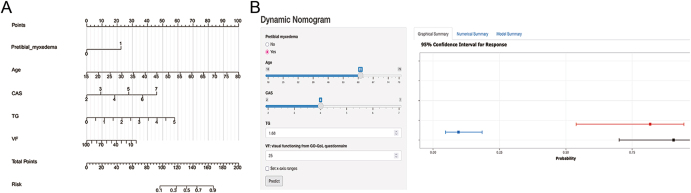
Prediction of the incidence of DON based on clinical characteristics and laboratory findings at the initial visit. (A) A graphical nomogram, derived from a multivariable model, is used to predict the likelihood of DON in patients with TED. Each variable in the model is assigned a score. The probability of DON occurrence in a population with these characteristics can be estimated by drawing a vertical line from the respective predictor values to the score scale at the top and summing the corresponding scores. The total points, obtained by manually summing the scores, correspond to the probability of DON. (B) A web-based probability calculator for DON (https://donnomo.shinyapps.io/dynnomapp/).

Multivariable logistic regression analysis identified five independent predictors of DON, namely age (odds ratio (OR) = 1.101; 95% confidence interval (CI) = 1.054–1.150; *P* < 0.001), PTM (OR = 4.085; 95% CI = 1.054–14.342; *P* = 0.028), TG levels (OR = 2.060; 95% CI = 1.277–3.322; *P* = 0.003), subjective VF score (OR = 0.980; 95% CI = 0.964–0.996; *P* = 0.014), and CAS (OR = 1.776; 95% CI = 1.234–2.557; *P* = 0.002) (Table 2). Based on these five predictive factors, a nomogram was constructed to estimate the likelihood of DON occurrence (Fig. 4A). In addition, a web-based diagnostic model was developed for clinical application and visualization (https://donnomo.shinyapps.io/dynnomapp/), enabling real-time DON probability estimation based on patient-specific data (Fig. 4B).

### Performance of the diagnosis model for DON

**Figure 5:**
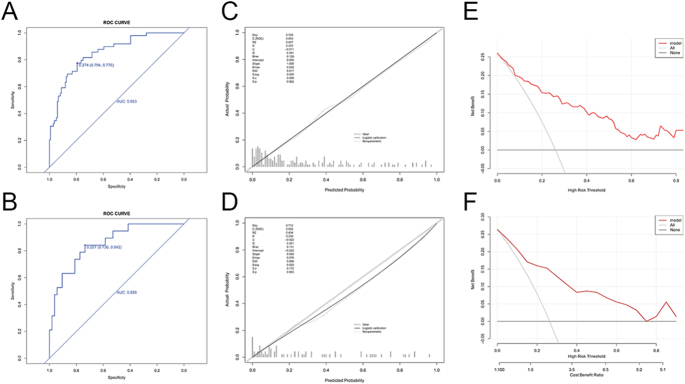
Performance of predictive model for DON. (A) Receiver operating characteristic (ROC) of the derivation cohort, (B) ROC of the validation cohort, (C) calibration curve of the derivation cohort, (D) calibration curve of the validation cohort, (E) decision curve of the derivation cohort, (F) decision curve of the validation cohort.

Performance evaluation of the final model demonstrated robust discrimination, with area under the curve (AUC) values of 0.853 (95% CI: 0.792–0.914) and 0.856 (95% CI: 0.762–0.950) in the derivation and validation cohorts, respectively (Fig. 5A and B). The Hosmer–Lemeshow test yielded *P*-values of 0.989 and 0.873 for the derivation and validation cohorts, respectively. The predicted probabilities of DON based on the nomogram and the actual outcomes were visualized through calibration curves (Fig. 5C and D). Notably, the nomogram exhibited strong calibration, with the predicted probabilities closely aligning with observed outcomes. Decision curve analysis (Fig. 5E and F) further supported the clinical utility of the model, demonstrating a favorable net benefit across a broad range of threshold probabilities. Collectively, these validation results confirm the reliability and potential applicability of the proposed model for DON risk assessment in clinical settings.

## Discussion

DON is a type of vision-threatening condition in TED, underscoring the importance of identifying its risk factors for early intervention. This study aimed to determine significant predictors of DON in patients with moderate-to-severe TED. Through LASSO and logistic regression analyses, age, CAS, TG levels, subjective VF score, and PTM were identified as key predictors of DON onset. Based on these findings, a diagnostic predictive model was developed for DON. The model, presented through a nomogram and a website, offers a practical DON risk prediction approach for clinicians, particularly endocrinologists. By integrating readily available clinical and serological parameters, this tool facilitates early identification of high-risk individuals, enabling timely intervention and enhanced patient management.

Among patients with moderate-to-severe TED, the incidence of DON (5–8%) is markedly higher than that in the general TED population (20, 21). Previously, the prevalence of DON in moderate-to-severe TED cases has been reported to range from 10.5 to 42.9% (10, 12, 13). In the institutional cohort of the present study, DON prevalence among hospitalized patients with TED was found to be 26.5%, which was attributed to the inclusion of patients with either moderate-to-severe TED or DON. Early therapeutic intervention, particularly within 6 weeks of symptom onset, has been shown to notably improve clinical outcomes. Therefore, systematic risk stratification of patients with TED and vigilant monitoring of high-risk cases are essential.

Advancing age has been strongly associated with increased disease severity in TED and has been identified as one of the most influential risk factors for DON in both Western and Eastern populations (22, 23). Herein, the mean age of patients with DON was 58 years, compared with 48 years for those without DON. These results are consistent with previous findings and confirm advanced age as an independent risk factor for DON onset (13, 24). Furthermore, a significant negative linear correlation was observed between age and the degree of proptosis in Chinese patients (*ρ* = −0.317). Notably, younger individuals tend to exhibit orbital fat expansion, resulting in more pronounced proptosis. Conversely, older patients often demonstrate extraocular muscle expansion near the posterior orbital rim, predisposing them to orbital apex crowding and limited proptosis (25).

CAS, a widely recognized quantitative index of TED activity, has been consistently associated with the risk of optic nerve involvement (26, 27). Many previous studies have combined CAS with additional parameters, such as the Hardy–Rand–Rittler color vision test, retinal nerve fiber layer thickness, and various metabolic and inflammatory biomarkers, to enhance the predictive accuracy for DON (28, 29). Consistent with these studies, the present study demonstrated that the baseline CAS values in patients with DON were significantly higher compared with those in individuals without DON. Multivariate logistic regression analysis further confirmed CAS as a critical and independent predictor for diagnosing DON.

To the best of our knowledge, this is the first study to identify PTM as a significant factor in DON onset. Multivariate logistic regression analysis indicated that patients with both TED and PTM had a 4.085-fold increased likelihood of developing DON. This association was attributed to the shared TRAb-mediated autoimmune mechanisms and fibroblast activation, which contribute to both orbital and dermal manifestations. Recent evidence shows that teprotumumab, an insulin-like growth factor 1 receptor inhibitor, induces concurrent regression of TED and PTM, further supporting the hypothesis of a common underlying pathophysiological mechanism (30).

Many studies have associated TED activity with TC and LDL-C levels (31, 32). However, the specific contribution of these lipid variables to DON pathogenesis remains unclear. This study provides novel evidence that elevated TG and LDL-C levels are potential risk factors for DON development, with TG being identified as an independent predictor in the developed diagnostic model. These findings are consistent with previous studies indicating that patients with diabetes exhibit an increased susceptibility to DON (11, 13, 33). This may be attributed to the microvascular damage, heightened inflammatory responses, and advancing age of patients with diabetes. Although multivariate analysis did not reveal significant collinearity between diabetes and other variables (such as age and TG levels), their potential interactions may have attenuated the statistical significance of diabetes in the model. Nevertheless, diabetes remains a clinically relevant contributor to DON risk.

The GO-QoL subjective VF score in patients with DON was found to be significantly lower than that in patients with moderate-to-severe TED, showing a strong negative correlation with the Gorman score. Subjective VF score emerged as a key indicator of disease burden in TED patients, and we incorporated it into our diagnostic model. Thyroid-stimulating immunoglobulin and TRAb have been implicated in both TED activity and proptosis (34, 35). Herein, TRAb levels exhibited a statistically significant positive correlation with CAS (*ρ* = 0.228), suggesting a contributory role of TRAb in driving inflammatory activity. However, no association was observed between TRAb levels and DON occurrence, which is consistent with previous studies (10, 13).

This study has some limitations. First, the data were obtained from a single center, which possibly introduced some confounding bias. Second, because the cohort included only hospitalized patients with moderate-to-severe TED and DON, the observed DON incidence was higher than that reported for general TED populations. Mild patients at our center mostly opt for local treatment; therefore, the exclusion of 12 mild patients was due to their strong preference for inpatient treatment. Consequently, the derived diagnostic model may exhibit limited accuracy when applied to cases with mild severity, presenting a challenge for broader clinical generalizability. Finally, owing to the cross-sectional design of the study, associations were established between the investigated factors and DON rather than the establishment of causal relationships. Future research needs to focus on including prospective, multicenter studies to validate the present findings and establish causality, along with conducting external validation of the nomogram across diverse clinical populations. In addition, longitudinal data should be incorporated to better characterize the temporal relationships between risk factors and DON development.

DON diagnosis primarily relies on advanced imaging modalities such as CT, MRI, OCT, and electroretinography (ERG), which assess orbital fat infiltration, optic nerve sheath morphology, and cerebrospinal fluid (CSF) dynamics (36, 37, 38). Although these techniques are informative, they require substantial healthcare resources and complex multidisciplinary involvement, limiting their accessibility in routine clinical settings. To address these constraints, we developed and validated a rapid diagnostic model for DON based on routine clinical and serological data. The model demonstrated strong predictive performance, with area under the curve (AUC) values of 0.853 in the derivation cohort and 0.856 in the validation cohort, indicating high reliability and potential for clinical integration.

In conclusion, the findings of this study identify advanced age, high CAS, PTM presence, lower subjective VF score, and elevated TG levels as significant independent predictors of DON. The novel prediction model was established and internally validated after incorporating these clinically accessible parameters. The proposed risk stratification tool can enable non-specialists to effectively identify patients with TED who are at high risk for DON development and require urgent specialist evaluation in primary care settings. Based on the findings of the prediction model, we recommend immediate ophthalmological referral for patients classified as high-risk for timely intervention.

## Supplementary materials



## Declaration of interest

The authors declare that there is no conflict of interest that could be perceived as prejudicing the impartiality of the research reported.

## Funding

This research was supported by National Natural and Science Foundation of China Grant (Grant No. 82270833) awarded to YLi. The other authors have no funding information to declare.

## Author contribution statement

RH conceived and designed the study, performed data analysis and interpretation, and wrote the manuscript. ST, XL, ZL, ZC, JC, YW, and FJ contributed to data acquisition, analysis, and interpretation. JZ, C Wan, YS, LS, ZW, CL, XY, C Wang, WW, Y Lai, YC, and XW contributed to resources and data curation. Y Li, Z S, and WT provided guidance throughout the study, supervising the research and critically revising the manuscript for important intellectual content. All authors reviewed and approved the final manuscript.
