# Supplementary material for: Predicting dysthyroid optic neuropathy in moderate-to-severe thyroid eye disease: a clinically applicable nomogram
Source: Eur Thyroid J. 2025 Dec 18;14(6):e250226. doi: 10.1530/ETJ-25-0226 (PMC12720399; doi:10.1530/ETJ-25-0226)
Supplement: Supplementary file 1 [file supplementary_materials.pdf]

Supplementary Table S1. Thyroid Disease Status Among Patients in the Derivation Cohort

	Total n=185	DON n=49	Moderate to severe TED n=136	*P-Value*
Duration of thyroid disease (Month)-median (IQR)	10.0 (4.0-22.0)	10.0 (4.5-21.5)	10.0 (4.0-22.0)	0.619[b]
Thyroid disease-n (%)				
Graves Disease	172 (93.0)	43 (87.8)	129 (94.9)	0.098[d]
Hypothyroidism	6 (3.2)	4 (8.2)	2 (1.5)	
Hashimoto's disease	5(2.7)	1 (2.0)	4 (2.9)	
Euthyroid TED	2 (1.1)	1 (2.0)	1 (0.7)	
FT4 (pmol/L)-median (IQR)	12.82 (10.72-14.93)	13.55 (11.29-15.70)	12.64 (10.69-4.53)	0.110 [b]
FT3 (pmol/L)-median (IQR)	4.55 (3.98-5.43)	4.51 (3.89-5.94)	4.56 (4.06-5.30)	0.953 [b]
TSH (mIU/L)-median (IQR)	0.35 (0.01-2.31)	0.27 (0.003-1.711)	0.68 (0.01-3.00)	0.121 [b]
TPOAb (IU/mL)-median (IQR)	14.04 (0.99-236.85)	32.29 (0.82-472.47)	10.50 (1.03-209.72)	0.374 [b]
TGAb (IU/mL)-median (IQR)	2.28 (1.30-31.26)	2.24 (1.49-156.68)	2.34 (1.16-20.00)	0.316[b]
Vitamin D3 (ng/mL)-median (IQR)	18.00 (13.67-24.52)	16.90 (13.00-23.78)	18.39 (13.85-25.12)	0.306 [b]

The reference ranges for the laboratory tests were as follows: FT3: 2.43–6.01 pmol/L; FT4: 9.01–19.05 pmol/L; TSH: 0.35–4.94 mIU/L; TPOAb: 0.00–5.61 IU/mL; TGAb: 0.00–4.11 IU/mL; Vitamin D3: ≤20 ng/mL (vitamin D deficiency); 20–30 ng/mL (vitamin D insufficiency); and ≥30 ng/mL (normal vitamin D levels).

Statistical analysis was performed using: a) Student's t-test; b) Mann-Whitney U test; c) Chi-square test or corrected Chi-square test; and d) Fisher's exact test.

Statistically significant p-values are presented in bold

Supplementary Table S2. Demographics And Clinical Characteristics Of All Patients

	Total n=257	Derivation cohort n=185	Validation cohort n=72	*P-value*
Age (years)- mean ± SD	50.81±12.04	50.65±11.67	51.22±12.99	0.732[a]
DON-n (%)	68 (26.5)	49 (26.5)	19 (26.4)	0.987[c]
Sex-n (%)				
Female	157 (61.1)	109 (58.9)	48 (66.7)	0.253[c]
Male	100 (38.9)	76 (41.1)	24 (33.3)	
BMI (kg/m^2)-median (IQR)	24.22 (22.03-26.87)	24.05 (22.05-27.17)	24.43 (21.48-26.35)	0.388[b]
Smoking history-n (%)				
No	155 (60.3)	112 (60.5)	43 (59.7)	0.904[c]
Yes	102 (39.7)	73 (39.5)	29 (40.3)	
Duration of thyroid disease (Month)-median (IQR)	10 (4-24)	10 (4-22)	11(4.25-25.5)	0.600[b]
Thyroid disease-n (%)				
Graves Disease	238 (92.6)	172 (93.0)	66 (91.7)	0.766[d]
Hypothyroidism	10 (3.9)	6 (3.2)	4 (5.6)	
Hashimoto's disease	6 (2.3)	5 (2.7)	1 (1.4)	
Euthyroid TED	3 (1.2)	2 (0.8)	1 (1.4)	
Treatment of thyroid disease-n (%)				
Antithyroid drugs	217 (84.4)	153 (82.7)	64 (88.9)	0.367[c]
Levothyroxine	20 (7.8)	15 (8.1)	5 (6.9)	
Without medication	20 (7.8)	17 (9.2)	3 (4.2)	
Thyroidectomy-n (%)	15 (5.8)	11 (5.9)	4 (5.6)	0.905[c]
Radioactive iodine (RAI)-n (%)	27(10.5)	23 (12.4)	4 (5.6)	0.106[c]
Family history of thyroid disease-n (%)	30 (11.7)	21 (11.4)	9 (12.5)	0.797[c]
Pretibial myxedema-n (%)	22 (8.6)	19 (10.3)	3 (4.2)	0.116[c]
Diabetes mellitus-n (%)	29 (11.3)	19 (10.3)	10 (13.9)	0.410[c]
Osteoporosis-n (%)	25 (9.7)	20 (10.8)	5 (6.9)	0.348[c]
Dyslipidemia-n (%)	105 (40.9)	74 (40.0)	31 (43.1)	0.655[c]
Duration of TED(Month)-median (IQR)	6 (3-12)	7 (4-12)	5.5 (3-12)	0.183[b]
CAS-median (IQR)	4 (3-5)	4 (3-5)	4 (3-5)	0.675[b]
Diplopia-n (%)				
Yes	163 (63.4)	121 (65.4)	42 (58.3)	0.290[c]
No	94 (36.6)	64 (34.6)	30 (41.7)	
Gorman score-n (%)				
No diplopia (0 points)	94 (36.6)	64 (34.6)	30 (41.7)	0.149 [c]
Intermittent diplopia (1 point)	58 (22.6)	45 (24.3)	13 (18.1)	

Inconstant diplopia (2 points)	41 (16.0)	34 (18.4)	7 (9.7)	
Constant diplopia (3 points)	64 (24.9)	42 (22.7)	22 (30.6)	
Proptosis (mm)-median (IQR)	21.0 (19.0-23.0)	21.0 (19.0-23.0)	22.0 (19.0-24.0)	0.214[b]
GO-QoL				
VF-median (IQR)	50.00 (37.50-75.00)	56.25 (37.50-75.00)	50.00 (37.50-75.00)	0.422 [b]
AP-median (IQR)	56.25 (43.75-81.25)	56.25 (43.75-81.25)	56.25 (43.75-81.25)	0.297 [b]
FT4 (pmol/L)-median (IQR)	12.76 (10.93-14.83)	12.82 (10.73-14.93)	12.63 (11.05-14.63)	0.842[b]
FT3 (pmol/L)-median (IQR)	4.49 (3.96-5.26)	4.55 (3.98-5.43)	4.32 (3.93-4.95)	0.057[b]
TSH (mIU/L)-median (IQR)	0.51 (0.01-2.31)	0.35 (0.01-2.31)	0.66 (0.09-2.35)	0.256[b]
TPOAb (IU/mL)-median (IQR)	17.63 (1.01-338.05)	14.04 (0.99-236.85)	55.20 (1.50-605.28)	0.153[b]
TGAb (IU/mL)-median (IQR)	2.64 (1.36-52.90)	2.28 (1.30-31.26)	5.05 (1.50-232.68)	0.084[b]
TRAb (IU/L)-median (IQR)	10.38 (4.29-22.40)	10.65 (4.18-22.52)	9.87 (4.87-19.14)	0.861[b]
TG (mmol/L)-median (IQR)	1.21 (0.89-1.79)	1.20 (0.85-1.83)	1.26 (0.97-1.74)	0.470[b]
TC (mmol/L)-median (IQR)	4.80 (4.08-5.57)	4.81 (3.98-5.60)	4.79 (4.18-5.34)	0.582[b]
LDLC (mmol/L)-median (IQR)	2.89 (2.38-3.54)	2.88 (2.37-3.51)	2.89 (2.39-3.56)	0.604[b]
Vitamin D3 (ng/mL)-median (IQR)	18.38 (13.80-24.58)	18.00 (13.67-24.52)	19.20 (14.10-25.18)	0.583[b]

Abbreviations: IQR interquartile range, SD standard deviation, TED thyroid eye disease, FT4 free thyroxine, FT3 free triiodothyronine, TSH thyroid-stimulating hormone, TPOAb thyroid peroxidase antibody, TGAb thyroglobulin antibody, TRAb TSH receptor antibody, TC total cholesterol, TG triglycerides, LDLC low-density lipoprotein cholesterol, GO-QoL Graves Orbitopathy on quality of life, VF subjective VF score (from GO-QoL), AP GO-QoL appearance score

Statistical analysis was performed using: a) Student's t-test; b) Mann-Whitney U test; c) Chi-square test or corrected Chi-square test; and d) Fisher's exact test.

Statistically significant p-values are presented in bold

Supplementary Table S3. Clinical Characteristics Of The Validation Cohort

	Total n=72	DON n=19	Moderate to severe TED n=53	*P-value*
Age (years)- mean ± SD	51.22±12.99	60.63±11.01	47.85±12.03	**<0.001[a]**
Sex-n (%)				
Female	48 (66.7)	14 (73.7)	34 (64.2)	0.449[c]
Male	24 (33.3)	5 (26.3)	19 (35.8)	
BMI (kg/m^2)-mean ± SD	24.19±3.42	23.80±3.90	24.33±3.25	0.281[a]
Smoking history-n (%)				
No	43 (59.7)	11 (57.9)	32 (60.4)	0.850[c]
Yes	29 (40.3)	8 (42.1)	21 (39.6)	
Duration of thyroid disease (Month)-median (IQR)	11.0 (4.25-25.5)	12.0 (6.0-24.0)	10.0 (4.0-28.5)	0.833[b]
Thyroid disease-n (%)				
Graves Disease	66 (91.7)	16 (84.2)	50 (94.3)	0.118[d]
Hypothyroidism	4 (5.6)	3 (15.8)	1 (1.9)	
Hashimoto's disease	1 (1.4)	0	1 (1.9)	
Euthyroid TED	1 (1.4)	0	1 (1.9)	
Treatment of thyroid disease-n (%)				
Antithyroid drugs	64 (88.9)	16 (84.2)	48 (90.6)	0.202[d]
Levothyroxine	5 (6.9)	3 (18.8)	2 (3.8)	
Without medication	3 (4.2)	0	3 (5.7)	
Thyroidectomy-n (%)	4 (5.6)	2 (10.5)	2 (3.8)	0.283[d]
Radioactive iodine (RAI)-n (%)	4 (5.6)	1 (5.3)	3 (5.7)	1.000[d]
Family history of thyroid disease-n (%)	9 (12.5)	1 (5.3)	8 (15.1)	0.479[c]
Pretibial myxedema-n (%)	3 (4.2)	1 (5.3)	2 (3.8)	1.000[d]
Diabetes mellitus-n (%)	10 (13.9)	4 (21.1)	6 (11.3)	0.506[c]
Osteoporosis -n (%)	5 (6.9)	1 (5.3)	4 (7.5)	1.000[d]
Dyslipidemia -n (%)	31 (43.1)	9 (47.4)	22 (41.5)	0.658[c]
Duration of TED(Month)-median (IQR)	5.5 (3.0-12.0)	6.0 (3.0-12.0)	5.0 (3.0-12.0)	0.918[b]
CAS-median (IQR)	4.0 (3.0-5.0)	4.0 (4.0-5.0)	3.0 (3.0-4.5)	**<0.001[b]**
Diplopia-n (%)				
Yes	42 (58.3)	13 (68.4)	29 (54.7)	0.299[c]
No	30 (41.7)	6 (31.6)	24 (45.3)	
Gorman score-n (%)				
No diplopia (0 points)	30 (41.7)	6 (31.6)	24 (45.3)	0.319[c]
Intermittent diplopia (1 point)	13 (18.1)	2 (10.5)	11 (20.8)	

Inconstant diplopia (2 points)	7 (9.7)	3 (15.8)	4 (7.5)	
Constant diplopia (3 points)	22 (30.6)	8 (42.1)	14 (26.4)	
Proptosis (mm)-mean ± SD	21.61±3.42	21.89±4.41	21.51±3.03	0.728[a]
GO-QoL				
VF-median (IQR)	50.00 (37.50-75.00)	37.50 (25.00-50.00)	56.25 (43.75-77.50)	**0.001[b]**
AP-median (IQR)	56.25 (43.75-81.25)	56.25 (43.75-81.25)	50.00 (43.75-81.25)	0.328[b]
FT4 (pmol/L)- mean ± SD	12.92±3.69	13.26±3.15	12.80±3.89	0.645[a]
FT3 (pmol/L)-median (IQR)	4.32 (3.93-4.95)	4.41 (4.01-5.40)	4.30 (3.88-4.89)	0.316[b]
TSH (mIU/L)-median (IQR)	0.66 (0.09-2.35)	0.40 (0.08-1.48)	0.98 (0.11-2.41)	0.368[b]
TPOAb (IU/mL)-median (IQR)	55.20 (1.50-605.28)	185.22(0.94-1000.00)	40.40 (1.56-486.05)	0.394[b]
TGAb (IU/mL)-median (IQR)	5.05 (1.50-232.68)	30.56 (1.09-620.96)	3.13 (1.51-67.92)	0.195[b]
TRAb (IU/L)-median (IQR)	9.87 (4.87-19.14)	16.06 (9.90-30.98)	7.35 (3.69-16.17)	**0.002[b]**
TG (mmol/L)-median (IQR)	1.26 (0.97-1.74)	1.43 (0.97-1.98)	1.22 (0.97-1.63)	0.222[b]
TC (mmol/L)-median (IQR)	4.79 (4.18-5.34)	4.72 (4.13-5.26)	4.79 (4.21-5.40)	0.730[b]
LDLC (mmol/L)-median (IQR)	2.89 (2.39-3.56)	2.83 (2.40-3.48)	2.89 (2.37-3.63)	0.808[b]
VitaminD3 (ng/mL)-median (IQR)	19.20 (14.10-25.18)	17.50 (13.50-22.80)	19.60 (14.20-26.15)	0.341[b]

Abbreviations: IQR interquartile range, SD standard deviation, TED thyroid eye disease, FT4 free thyroxine, FT3 free triiodothyronine, TSH thyroid-stimulating hormone, TPOAb thyroid peroxidase antibody, TGAb thyroglobulin antibody, TRAb TSH receptor antibody, TC total cholesterol, TG triglycerides, LDLC low-density lipoprotein cholesterol, GO-QoL Graves Orbitopathy on quality of life, VF subjective VF score (from GO-QoL), AP GO-QoL appearance score

Statistical analysis was performed using: a) Student's t-test; b) Mann-Whitney U test; c) Chi-square test or corrected Chi-square test; and d) Fisher's exact test.

Statistically significant p-values are presented in bold.
